# Success rate of naso-jejunal tube placement influenced by CRRT: possible removal of metoclopramide

**DOI:** 10.1186/s13054-021-03652-z

**Published:** 2021-06-30

**Authors:** Patrick M. Honore, Sebastien Redant, Thierry Preseau, Sofie Moorthamers, Keitiane Kaefer, Leonel Barreto Gutierrez, Rachid Attou, Andrea Gallerani, David De Bels

**Affiliations:** 1 ICU Dept, Brugmann University Hospital, ULB University, Brussels, Belgium; 2 Emergency Dept, Brugmann University Hospital, ULB University, Brussels, Belgium

**Keywords:** Naso-jejunal tube, CRRT, Metoclopramide elimination, CRRT interruption

With interest, we read the recent article by Wang et al. in which their logistic regression analysis identified the use of vasopressor agents, the presence of a neurological disease, high APACHE II and SOFA scores, an acute gastrointestinal injury (AGI) ≥ grade II, and the use of mechanical ventilation or continuous renal replacement therapy (CRRT) as independent risk factors influencing the success rate of placement of a naso-jejunal tube (NJT) [1]. The impact of all these conditions is easy to understand, except CRRT. We noted that the authors routinely administered 10 mg of metoclopramide has a molecular weight of 1,000 daltons (Da), before the procedure [2]. In CRRT, membranes with a cut-off value of 35,000 Da are routinely used, eliminating a large quantity of metoclopramide when administered [3]. New highly adsorptive membranes that can adsorb molecules with a molecular weight above 35,000 Da will increase this removal [4]. It is therefore reasonable to assume that only a very small portion of metoclopramide will be available to stimulate NJT migration in the patient while on CRRT. A simple method to avoid this, is to discontinue the CRRT one hour before administering the metoclopramide and to restart it when the NJT is in place. This is the same concept as CRRT interruption to obtain high peak/MIC ratio’s for antibiotics with concentration-dependent killing such as aminoglycosides, in a strategy to increase their bactericidal efficacy against resistant microorganisms [5].

## Data Availability

Not applicable.

## Abbreviations

AGI: Acute gastrointestinal
CRRT: Continuous renal replacement therapy
NJT: Naso-jejunal tube
Da: Daltons
MIC: Minimal inhibitory concentration
